# Aptamers as promising nanotheranostic tools in the COVID‐19 pandemic era

**DOI:** 10.1002/wnan.1785

**Published:** 2022-03-03

**Authors:** Christian K. O. Dzuvor, Ebenezer Larteh Tettey, Michael K. Danquah

**Affiliations:** ^1^ Bioengineering Laboratory, Department of Chemical and Biological Engineering Monash University Clayton Victoria Australia; ^2^ Department of Medical Laboratory Science University of Cape Coast Ghana; ^3^ Department of Chemical Engineering University of Tennessee Chattanooga Tennessee USA

**Keywords:** aptamers, bioaffinity, diagnostics, multivalent, nanomedicine, SARS‐COV‐2, therapeutics

## Abstract

The emergence of SARS‐COV‐2, the causative agent of new coronavirus disease (COVID‐19) has become a pandemic threat. Early and precise detection of the virus is vital for effective diagnosis and treatment. Various testing kits and assays, including nucleic acid detection methods, antigen tests, serological tests, and enzyme‐linked immunosorbent assay (ELISA), have been implemented or are being explored to detect the virus and/or characterize cellular and antibody responses to the infection. However, these approaches have inherent drawbacks such as nonspecificity, high cost, are characterized by long turnaround times for test results, and can be labor‐intensive. Also, the circulating SARS‐COV‐2 variant of concerns, reduced antibody sensitivity and/or neutralization, and possible antibody‐dependent enhancement (ADE) have warranted the search for alternative potent therapeutics. Aptamers, which are single‐stranded oligonucleotides, generated artificially by SELEX (Evolution of Ligands by Exponential Enrichment) may offer the capacity to generate high‐affinity neutralizers and/or bioprobes for monitoring relevant SARS‐COV‐2 and COVID‐19 biomarkers. This article reviews and discusses the prospects of implementing aptamers for rapid point‐of‐care detection and treatment of SARS‐COV‐2. We highlight other SARS‐COV‐2 targets (N protein, spike protein stem‐helix), SELEX augmented with competition assays and *in silico* technologies for rapid discovery and isolation of theranostic aptamers against COVID‐19 and future pandemics. It further provides an overview on site‐specific bioconjugation approaches, customizable molecular scaffolding strategies, and nanotechnology platforms to engineer these aptamers into ultrapotent blockers, multivalent therapeutics, and vaccines to boost both humoral and cellular immunity against the virus.

This article is categorized under:

Therapeutic Approaches and Drug Discovery > Emerging Technologies
Diagnostic Tools > Biosensing
Therapeutic Approaches and Drug Discovery > Nanomedicine for Infectious Disease
Therapeutic Approaches and Drug Discovery > Nanomedicine for Respiratory Disease

## Graphical abstract

The emergence of the COVID‐19 pandemic caused by SARS‐COV‐2 has resulted in a global public health threat, necessitating the need for low‐cost, easy‐to‐use, rapid, ultrasensitive, accurate point‐of‐care diagnostics, and effective treatment. In this review, we discuss recent development on aptamers as alternative potential SARS‐COV‐2 diagnostics and therapeutics. We also highlight promising strategies and targets for rapidly discovering and engineering ultrapotent aptamer‐based inhibitors, drug candidates, and vaccines against SARS‐COV‐2 escape mutants.

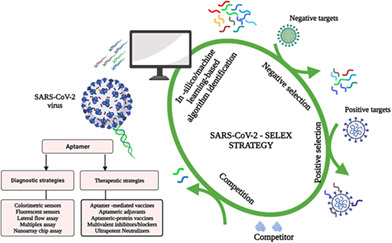

## 1 INTRODUCTION

Severe acute respiratory syndrome‐coronavirus‐2 (SARS‐COV‐2) is the pathogen that is currently causing a worldwide public health threat. The World Health Organization (WHO) named infection caused by the virus as coronavirus disease 2019 (COVID‐19). COVID‐19 started in China in a city called Wuhan, located in Hubei province. The disease begins with feverish symptoms, progresses to severe respiratory challenges and pneumonia‐like symptoms (Wrapp et al., 2020). According to WHO situational report‐70, as of October 28, 2021, a total of 245 million cases had been confirmed globally, with 4.97 million deaths. The mortality rate stands at 10% globally based on diagnosed cases (Overall mortality is lower). These have warranted urgent search for (i) rapid diagnostics for early disease detection, screening, and reduction of virus widespread (Vandenberg et al., 2021); (ii) effective therapeutics and prophylactics interventions against the virus (Chauhan et al., 2020); and (iii) effective and highly target‐specific vaccine candidates (Chung et al., 2020).

On the diagnostic front, various molecular, and antigen tests have been developed as gold standards to detect active COVID‐19 infections. While these tests are extraordinarily sensitive and provide point‐of‐care results, they suffer drawbacks such as lengthy and costly operational methods, longer turn‐around time, and require technical laboratory training (Carter et al., 2020). Also, various biologics such as neutralizing antibodies (Corti et al., 2021), nanobodies (Sasisekharan, 2021), and soluble Angiotensin‐converting enzyme (ACE2) decoys (Jing & Procko, 2021) have been employed to help control and treat the infection. Despite their demonstrated potencies, these biologics are faced with some challenges such as longer, costly, and/or intensive discovery and production methods (Gilchuk et al., 2020), reduced efficacies, and antigenic drift due to the emergence of mutational variants (Planas et al., 2021). Specifically, antibodies may induce antibody‐dependent enhancement (ADE) leading to increased viral infection and virulence in vivo (Lee et al., 2020). In addition, several SARS‐COV‐2 mutational variants have demonstrated reduced sensitivity to antibodies triggered by vaccines or infections (Corbett et al., 2021). Thus, countermeasures remain critical to help combat the prevailing COVID‐19 Infections on both fronts. Furthermore, advances in these countermeasures will enable future pandemic preparedness (Simpson et al., 2020). Due to their unique characteristics such as small size, high stability, low immunogenicity, advanced programmability, versatility, and safety, aptamers remain a great potential and intervention as diagnostics, prophylactics, and therapeutics against COVID‐19 and other future pandemics.

This comprehensive research review explicitly presents recent advances and prospects of aptamer‐based technologies for bioscreening and treatment of the SARS‐COV‐1 and SARS‐COV‐2.

With the rapid emergence of SARS‐COV‐2 variants of concerns, it has become imperative to probe into the design and development of potent inhibitors. Therefore, we suggest and/or propose artificial intelligence (AI) assisted methods for rapid aptamer discovery and interesting customizable nanotechnological and/or scaffold strategies to engineer aptamers into multivalent blockers/inhibitors, ultrapotent neutralizers, and highly immunogenic vaccines. This information will aid in the development of reliable point‐of‐care tests and effective therapeutics against COVID‐19 and other emerging infectious diseases. Finally, perspective on the aptamer isolation frontiers, mass production or development, sensitivity, standardization, and industrialization of aptasensors compared to other point‐of‐care diagnostics have been highlighted. These developed aptasensors will enable rapid testing in hospitals and airports, thus reducing lockdown and quarantine restrictions.

## 2 CORONAVIRUSES: GENOMICS, STRUCTURE, INTERACTIONS, AND INFECTION MECHANISM

**FIGURE 1:**
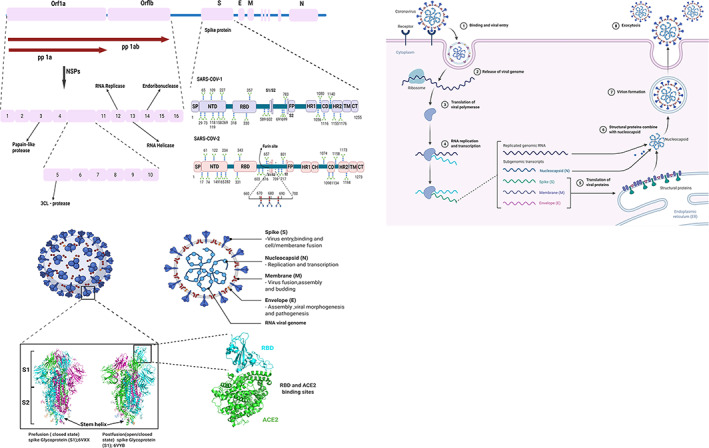
(a) Schematic representation of the SARS‐CoV‐2 genomic structure and multidomain structural juxtaposition of the SARS‐COV‐1 and SARS‐COV‐2 primary spike glycoprotein (not to scale). The region from ORF1a to ORF1b is expanded below to resolve nsp1–nsp16. The six accessory genes that encode six accessory proteins (orf3a, orf6, orf7a, orf7b, orf8, and orf10) are not shown. ORFs, open reading frame genes; S, spike protein gene; E, envelope protein gene; M, membrane protein gene; N, nucleocapsid protein gene. Schematic structure of the SARS‐COV‐2 spike glycoprotein domain consists of signal peptide (SP, 1–13), N‐terminal domain (NT,13–350) receptor‐binding domain (RBD, 319–541), furin protease cleavage sites (S_1_/S_2_), fusion peptide (FP, 788–806), heptad repeat 1 (HR1913–984))heptad repeat 2 central helix (CH, 987–1034), (HR2, 1163–1213) domain, transmembrane domain (TM, 1214–1237), and cytoplasmic terminal (CT, 1235–1273). The glycan and cleavage sites are depicted according to their position on the domains. (b) Schematic representation of the structure of SARS‐COV‐2 virus: Crystal structure**/**Cryo‐EM structure of Closed state (PDB: 6VXX) and open state (PDB: 6VYB) of SARS‐COV‐2 spike glycoprotein and ACE2‐spike protein interaction. (c) Simplified SARS‐COV‐2 ACE2 receptor‐mediated host cell entry, replication cycle, and exocytosis. Reproducible under Creative Commons Attribution license CC0 1.0)

The pathogen causing COVID‐19 has been identified to be similar to the virus that caused Severe Acute Respiratory Syndrome (SARS) in 2002, with about 800 people losing their life worldwide (Cho et al., 2011). The SARS coronavirus, SARS‐COV‐1 is an RNA virus that is positively stranded in nature with almost 30,000 nucleotides in the genome. Both SARS‐COV‐2 and SARS‐COV‐1 belong to the coronavirus family, specifically from the β‐coronavirus genera. A study by Xu et al. (2020) showed that the two viruses share almost the same gene sequence with 85% similarity, especially at the nucleotide level. Their genomic sequence containing six regions of difference (RD) partially code for both *orf* (open reading frame) lab, 1a, 1b, and gene sequences for the structural proteins (Figure 1a). These RDs serves as biomarkers for the virus identification and drug targets. The orf1a and orf1b constitute two‐thirds of the genome, which encodes 16 nonstructural proteins (NSP1–NSP16) within the pp1ab gene (Helmy et al., 2020). The NSPs are organized into a replication–transcription complex (RTC) for genome transcription and replication. For instance, NSP3 and NSP5 respectively encode for Papain‐like and 3CL proteases, which help in polypeptides cleavage and host innate immune response blockade (Alanagreh et al., 2020). The NSP12‐14 encode for RNA replicase, RNA helicase, and endoribonuclease, respectively. The remaining SARS CoV‐2 genes encode four structural proteins (S, M, E, and N), and six accessory proteins (orf3a, orf6, orf7a, orf7b, orf8, and orf10; Alanagreh et al., 2020).

The structural proteins consist of an inner nucleocapsid (N) surrounded by an outer layer of an envelope (E) and membrane_(M) proteins with glycoprotein spike (S) protruding from the surface (Wrapp et al., 2020; Figure 1a,b). Structural and functional analysis of the N, E, and S proteins indicated that SARS‐COV‐1 and SARS‐COV‐2 have significant similarities and differences, and the S and N proteins provide stability to the virus. The critical surface protein found in SARS‐COV‐2 and SARS‐COV‐1 differs and are only 75% identical, hence suggesting their mechanism to survival in the human host (Lu et al., 2020; Srinivasan et al., 2020). The Membrane (M) protein helps assemble and form the viral core and envelope via interaction with the N protein (Li et al., 2019). The envelope (E) protein is a transmembrane helix protein of 76–109 amino acids (Kuo et al., 2007). It comprises three domains; amino‐terminal, transmembrane, and carboxy‐terminal. The amino and carboxy‐terminal consist of a short and long hydrophilic peptide, respectively. The transmembrane part is hydrophobic in nature and consists of α‐secondary helix structure. The E protein is involved in host cell recognition and viral assembly (Tan et al., 2005). However, its function in coronavirus infection remains elusive. Hence, it is becoming challenging to consider this as an essential target for the development of therapeutics. Altogether, in antibody profile studies on SARS patients' sera, both M and E protein lack neutralizing or antagonistic antibodies as these proteins are located within the viral envelope (Tan et al., 2004). However, antagonist development for these M and N proteins would contribute to relevant alternative therapeutic treatments for coronavirus.

Coronavirus infection stimulates an immune response in the host using the N protein. During the early stage of the disease, the N protein is predominantly expressed, and this has been a target for diagnostics development (Che et al., 2004). It is an essential structural protein responsible for transcription and assembling of the viral particle. It is also involved in the formation of helical ribonucleoproteins, regulation of RNA synthesis, and transcription and metabolism modulation of infected cells (Tang et al., 2005). Primarily, the CoV N protein binds and packs the viral genomic RNA into a nucleocapsid protein complex. All these attributes and functionalities have been demonstrated in ex vivo and in vivo biochemical studies. It also possesses a binding affinity for nsp3 located on the replicase complex and the M protein (Fehr & Perlman, 2015), leading to a transition from viral genome to replicase transcriptase complex, and finally packaging of the genomic nucleocapsid into a viral particle. The N protein consists of two separate domains (Amino and carboxy‐terminus domain; McBride et al., 2014). These domains connected via a short Serine‐Arginine linker can bind RNA via the contrasting mechanism. The amino and carboxy terminus domain undergo RNA binding and oligomerization, respectively, and the linker is responsible for phosphorylation. The amino terminal‐genomic RNA binding is possible via electrostatic interactions. Also, relevant amino acids residues in the amino‐terminal domain are responsible for the viral RNA binding and infection (Grossoehme et al., 2009). These features of N protein have assisted in the development of diagnostic assays, several specific candidate antibodies and vaccines have emerged against SARS‐COV virus.

Both SARS‐COV‐2 and SARS‐COV‐1 N protein are 90% identical (Gralinski & Menachery, 2020), indicating the probability of having similar antibodies. SARS‐COV N protein can neutralize the host immune response, a characteristic that has not yet been identified in the SARS‐COV‐2 N protein (G. Li et al., 2020).

According to Lu et al. (2020), SARS‐COV‐1 S protein is shorter than SARS‐COV‐2 S protein. Despite this, the spike S protein stalk in both viruses is highly homologous with 99% identity (Chan, Kok, et al., 2020). In general, both SARS‐COV‐1 and SARS‐COV‐2 S glycoproteins are multidomain proteins consisting of 1255aa and 1273aa, respectively (Figure 1a).

The S glycoprotein, consisting of S1 and S2 units, is predicted to form α‐helical secondary coiled‐coil structure (Gui et al., 2017; Kirchdoerfer et al., 2016; Walls et al., 2020). Within the S1 domain, is a receptor‐binding domain (RBD) which recognizes and interacts directly with the human ACE2 receptor. S1 unit consists of N and C terminal domains (NTD, CTD). S2 helps in the virus‐cell membrane fusion and anchorage. The S2 part harbors other proteins such as putative fusion peptide and heptapeptide repeats (HR1, HR2) and stem helix domains. In the final stages of S2 protein‐mediated membrane fusion, the SARS‐COV peptide fold into anti‐parallel six‐helix bundles (Bosch et al., 2004; Zhu et al., 2004). The membrane‐anchored S2 subunit is supported and stabilized by the S1 unit. In SARS‐COV‐2, Both S1 and S2 subunits are separated by a furin cleavage site. Furin is a host protease that resides in the Golgi. The removal of this cleavage site affects the virus entry into VeroE6 and BHK cells. The spike protein can induce immune responses and neutralizes antibodies against virus infections, suggesting that therapeutics, if developed against S protein could induce certain antibodies. These antibodies may either inhibit the viral binding and fusion or neutralize the virus infection (Chen, Li, et al., 2020). A collection of these candidate therapeutics and vaccines for SARS‐CoV can be seen in this review article (Du et al., 2009).

From structure studies and biochemical tests (Letko et al., 2020; Walls et al., 2020; Wan et al., 2020; Wrapp et al., 2020), SARS‐COV‐2 S protein possesses a higher binding affinity to the host cell than the SARS‐COV‐1 S. This was ascribed to the mutation of amino acid residues located in the receptor binding domain (RBD) of the spike S protein. Also, the SARS‐COV‐2 S has a unique polybasic cleavage site and three adjacent predicted O‐linked glycans, which are unavailable in SARS‐COV‐1 S and related family B of betacoronaviruses. Both SARS‐COV‐1 and SARS‐COV‐2 possess N‐linked glycans across the entire S protein. The virus utilizes the spike glycan to shield and escape the immune system (Casalino et al., 2020).

The angiotensin‐converting enzyme 2 (ACE2) is the receptor in the human host cell that interacts with the S protein of COVs through affinity binding to facilitate infection (Hoffmann et al., 2020). The S protein is used by the virus for entry into the host cell and subsequently fusing into the cell membrane. These two steps are essential in viral pathogenesis and infection (Benvenuto et al., 2020). The ease of human‐to‐human spread of COVID‐19 has been associated with the spike protein having a high affinity for the ACE2 receptor (Chan, Yuan, et al., 2020). Figure 1c shows the schematic representation of ACE2 receptor interaction with SARS‐COV‐2 and its replication stages.

## 3 EMERGING IN VITRO DETECTION AND DIAGNOSTIC TESTS FOR COVID‐19

Many research institutes and/or companies have manufactured several COVID‐19 in vitro diagnostic kits, requiring approvals from the FDA and other regulatory bodies. These testing assays and kits differ in the biochemistry and turnaround time. Most of the new testing assays and kits developed to support COVID‐19 mitigation efforts have received Emergency Use Authorisation (EUA) from FDA and are limited to the laboratories that invented them.

Currently, the diagnostic tests approved for the detection of SARS‐COV‐2, and in fact, COVs in general, include molecular tests such as reverse‐transcription polymerase chain reaction (RT‐PCR), isothermal nucleic acid amplification, Loop‐mediated isothermal amplification (LAMP), next‐generation sequencing (NGS), blood‐based serological test, and antigen test. Table 1 shows the list of some approved products and tests published by the FDA. We refer readers to recent extensive reviews (Feng et al., 2020; Udugama et al., 2020) for other FDA‐approved commercially available products as it is outside the scope of this review. To the best of our knowledge, over 900 COVID‐19 test and collection kits have been developed, among which the FDA has authorized 400 consisting of 235 molecular, 88 antibody, and 34 antigen tests.

**TABLE 1:** Some FDA approved diagnostic test kits for COVID‐19 infections

Product	Manufacturers	Test type	Result time (h)	Approval status
Real‐time SARS‐COV‐2	Abbott	PCR	4–6	FDA‐EUA
ID NOW COVID‐19 test	Abbott	Isothermal amp‐PoC	<1	FDA‐EUA
AvellinoCoV2	Avellino Labs	PCR	24–48	FDA‐EUA
BioGX SARS‐COV‐2 reagents	BioGX,BD	PCR	2–3	FDA‐EUA
Real‐time fluorescent RT‐PCR kit	BGI	PCR	3	FDA‐EUA
BIOFIRE COVID‐19 test	BioMerieux‐BioFire Defense	PCR	<1	FDA‐EUA
2019‐nCoV real‐time RT‐PCR Dx panel	CDC	PCR	24–72	FDA‐EUA
qSARS‐CoV‐2IgG/IgM rapid test kit	Cellex	Serological	<1	FDA‐EUA
COVID‐19 ELISA IgG antibody test	Mount Sinai Laboratory	Serological	<1	FDA‐EUA
DPP COVID‐19 IgM/IgG system	Chembio Diagnostic System, Inc	Serological	<1	FDA‐EUA
VITROS immunodiagnostic products anti‐SARS‐COV‐2 total reagent pack	Ortho Clinical Diagnostic, Inc	Serological	<1	FDA‐EUA
Xpert Xpress SARS‐COV‐2 test	Cepheid	PCR‐PoC	<1	FDA‐EUA
Logix smart Coronavirus COVID‐19 test	Co‐Diagnostics	PCR	1–2	FDA‐EUA
Simplexa COVID‐19 direct	DiaSorin Molecular	PCR	1	FDA‐EUA
ePlex SARS‐COV‐2 test	GenMark Diagnostics	PCR	2	FDA‐EUA
COVID‐19 RT‐digital PCR detection kit	Gnomegen	PCR	4–6	FDA‐EUA
Panther fusion SARS‐COV‐2 Assay	Hologic	PCR	3	FDA‐EUA
Smart dectect SARS‐COV‐2rRT‐PCR kit	InBios International	PCR	4–6	FDA‐EUA
CoV‐19 IDx assay	Ipsum Diagnostics	PCR	24	FDA‐EUA
Covid‐19 RT‐PCR test	LabCorp	PCR	24	FDA‐EUA
ARISES SARS‐COV‐2 assay	Luminex Molecular Diagnostics	PCR	2	FDA‐EUA
NxTAG CoV extended panel assay	Luminex Molecular Diagnostics	PCR	4	FDA‐EUA
Accula SARS‐COV‐2 test	Mesa Biotech	PCR‐PoC	<1	FDA‐EUA
SARS‐COV‐2 assay, 288/96 molecular system	NeuMoDx	PCR	1–2	FDA‐EUA
New coronavirus RT‐PCR test	Perkin Elmer	PCR	4–6	FDA‐EUA
COVID‐19 genesing real‐time PCR assay	Primerdesign	PCR	2	FDA‐EUA
QIAstat‐Dx respiratory SARS‐CoV‐2 panel	Qiagen (acq.by Thermo Fisher)	PCR	96–120	FDA‐EUA
Quest SARS‐COV‐2rRT‐PCR	Ouest	PCR	1	FDA‐EUA
Lyrra SARS‐COV‐2 assay	Quidel	PCR	4–6	FDA‐EUA
Cobas SARS‐COV‐2 test	Roche	PCR	3–8	FDA‐EUA
SARS‐COV‐2 RTqPCR detection kit	ScienceCell Research Labs	PCR	4–6	FDA‐EUA
TaqPath COVID‐19 combo kit	Thermo Fisher	PCR	4	FDA‐EUA
NY SARS‐COV‐2 real‐time RT‐PCR	Wadsworth Center,NY State Dept of Public Health	PCR	24–72	FDA‐EUA
SARS‐COV‐2 + influenza A & B RT‐qPCR kit	3D Medicines	PCR	4–6	CE Mark
Real quality RQ‐2019‐nCoV	AB ANALITICA	PCR	4–6	CE Mark
Bosphore 2019‐nCoV detection kit	Anatolia Geneworks	PCR	2	CE Mark
SARS‐COV‐2, influenza, RSV panel	AusDiagnostics	PCR	4–6	CE Mark
AccuPower COVID 19 real‐time RT‐PCR kit	Bioneer	PCR	8	CE Mark
Q‐Sens 2019‐nCoV detection kit	Cancer Rop	PCR	2	CE Mark
VIASURE SARS‐COV‐2 real‐timePCR	CerTest Bioter,BD	PCR	3	CE Mark
VitaPCR SARS‐COV2 assay	Credo Diagnostics Biomedical	PCR‐PoC	<1	CE Mark
QuantiVirus SARS‐COV‐2 test	DiaCarta	PCR	2	CE Mark
EasyScreen SARS‐COV‐2 Detection kit	Genetic Signature	PCR	4–5	CE Mark
Detection kit for SARS‐CoV‐2	Genetic Health	PCR	4	CE Mark
qCOVID‐19, CLART COVID‐19	Genomica /PharmMar Group	PCR	5	CE Mark
2019 real‐time PCR Kit	Kogene Biotech	PCR	4–6	CE Mark
GeneFinder COVID‐19 RealAMp kit	OsangHealth	PCR	4–6	CE Mark
Allplex 2019‐nCoV assay	Seegene	PCR	4	CE Mark
DiaPlex Q 2019‐nCoV detection kit	SoIGent	PCR	2	CE Mark
SARS‐COV‐2 clinical sequence assay	Vision Medicals	NGS	>12	CE Mark
Multiple real‐time PCR kit	Beijjing Applied Biological Technologies (XABT	PCR	4–6	CE Mark
SARS‐COV‐2RT‐PCR test	Children Hospital of Philadelphia (CHOP)	PCR	4–6	LDT (EUA)
MGH COVID‐19qPCR assay	Massachuesetts General Hospital	PCR	4–6	LDT (EUA)
SARS‐COV‐2 assay	Northwestern Medicine	PCR	4–6	LDT (EUA)
Viracor SARS‐COV‐2 assay	Viracor Eurofins Clinical Diagnostics	PCR	4–6	LDT (EUA)
Applied Biosystems TaqPath COVID‐19 combo kit	Rutgers Clinical Genomics Laboratory	PCR	4–6	LDT (EUA)
SDI SARS‐CoV‐2 assay	Special Diagnostic Laboratories	PCR	4–6	LDT (EUA)
UNC Health SARS‐COV‐2 real‐time RT‐PCR test	University of North Carolina	PCR	4–6	LDT (EUA)
Stanford SARS‐COV‐2 assay	Standard Health care	PCR	4–6	LDT (EUA)
Orig3n 2019 novel coronavirus (COVID‐19) test	Orig3n, Inc.	PCR	4–6	LDT (EUA)
SARS‐COV‐2 PCR test	Yale new Haven Hospital	PCR	4–6	LDT (EUA)
CDI enhanced COVID‐19 test	Hackensack University Medical Centre	PCR	4–6	LDT (EUA)
CirrusDx SARS‐COV‐2 assay	CirrusDx Laboratories	PCR	4–6	LDT (EUA)
Childrens‐Altona‐SARS‐COV‐2 assay	Infectious Diseases Diagnostics	PCR	4–6	LDT (EUA)
SARS‐COV‐2 test	Exact Sciences Laboratories	PCR	4–6	LDT (EUA)
SARS‐COV‐2 test	Integrity Laboratories	PCR	4–6	LDT (EUA)
COVID‐19 RT‐PC test	Medicine Lab of Baptist Hospital	PCR	4–6	LDT (EUA)
Explify respiratory	IDbyDNA	NGS	24–48	LDT
COVID‐19 home test kits	Carbon Health	PCR	72–144	Discontinued
At‐home Covid‐19 test	Everlywell	PCR	48	Discontinued
Covid‐19 home test kit	Nurx Molecular Testing Labs	PCR	48	Discontinued
GenBody COVID‐19 Ag	GenBody Inc	Lateral flow	<1	EUA
QuickVue at‐home COVID‐19 Test	Quidel Corporation	Lateral flow	<1	EUA
Flowflex COVID‐19 antigen home test	ACON Laboratories, Inc	Lateral flow	<1	EUA
BinaxNOW COVID‐19 antigen self‐test	Abbott Diagnostics Scarborough, Inc	Lateral flow	<1	EUA
BD veritor system	Becton, Dickinson and Company (BD)	Immunochromatographic	<1	EUA
QIAreach SARS‐COV‐2 antigen test	QIAGEN GmbH	Lateral flow	<1	EUA
LIAISON SARS‐CoV‐2 Ag	DiaSorin, Inc.	Immunochromatographic	<1	EUA
CareStart COVID‐19 antigen	Access Bio, Inc	Lateral flow	<1	EUA

*Note*: Available on https://www.fda.gov/medical‐devices/emergency‐situations‐medical‐devices/emergency‐use‐authorizations, accessed on October 31, 2021.

Abbreviations: EUA, emergency use authorization; FDA, Food and Drug Authority; LDT, laboratory‐based test; NGS, next‐generation sequencing; PCR, polymerase chain reaction; PoC, Point‐of‐Care.

**FIGURE 2:**
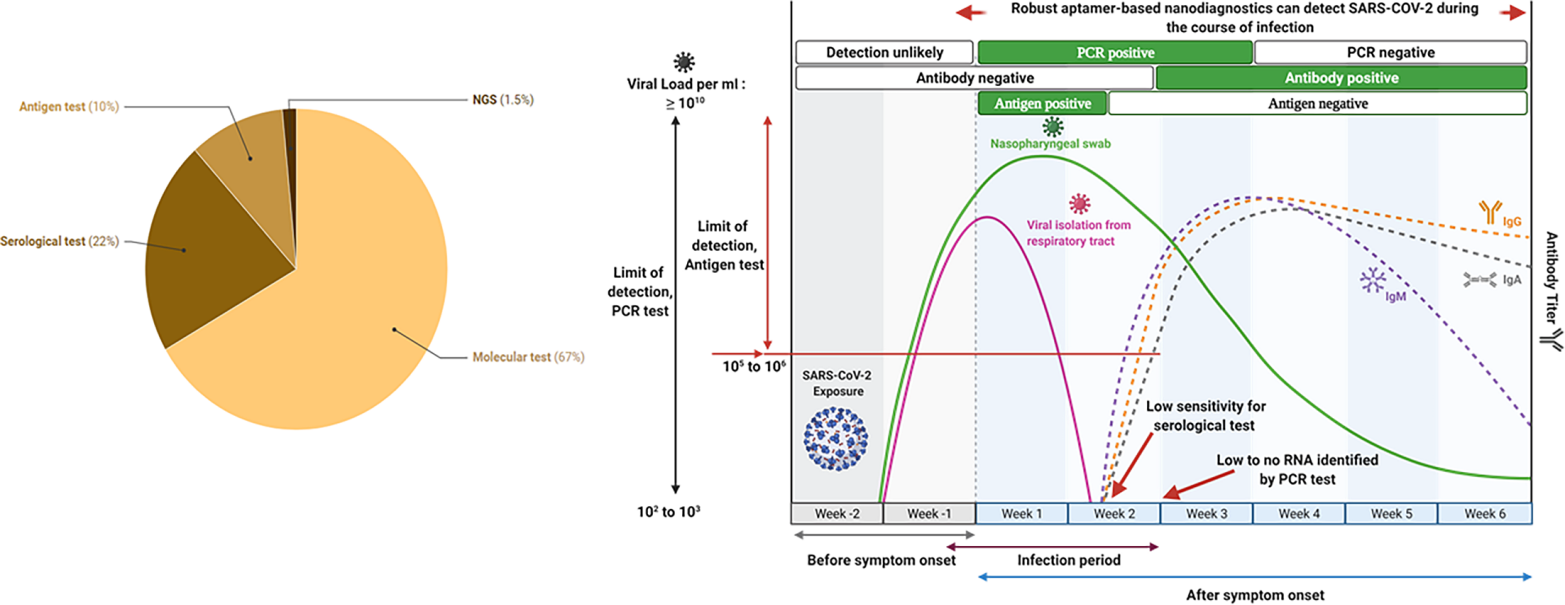
(a) Distribution of diagnostic tests and kits based on techniques or test type. Figure created with *Datawrapper*. (b) Schematic representation of the timeline of COVID‐19 infection and test positivity by either PCR‐based, antigen‐test, serological (antibody titer) testing, and aptamer‐based assay/test. Notably, both PCR and antigen tests are positive earlier in the disease course, while serological tests are positive later in the disease course. Aptamer‐based diagnostics can detect the virus during the course of infection

Most of these tests and kits are based on the Polymerase Chain Reaction (67%; Figure 2a).

In this technique (Udugama et al., 2020), RNA from inactivated viruses is extracted and purified from a nasopharyngeal swab collected from the patient. The purified RNA is then reverse transcribed and amplified to DNA. Through repeated heating and cooling, millions of copies of DNA are made. These viruses' DNAs are then mixed with fluorescent dyes, which give off more light after binding to the virus DNA, an indication of the presence of the virus in the collected sample.

The ease of use, specificity, and high sensitivity of RT‐PCR make it an effective method for SARS‐COV‐2 detection. It detects the virus from sputum, blood, urine, saliva, pharyngeal swabs, nasal, anal swabs, and stool specimen. RT‐PCR kits may have three different assays, with each associated with a different SARS‐COV‐2 gene target. Hence, the probability of false negatives result is minimal due to triplex amplification. The RT‐PCR has been shown to detect 4–8 viral copies per swab or source by amplifying targets in Orf1ab, E, and N viral genes at a 95% confidence level for each target sequence (Lin et al., 2020). However, RT‐PCR identified 47%–59% of the positive cases in COV tests, and 75% of results initially indicated as negative were found positive on the repeated run (Ai et al., 2020; Xie et al., 2020). Another shortfall of RT‐PCR assay relates to heat inactivation of the samples, and this may lead to inactivation of the viral particle, hindering practical downstream diagnostic evaluation (C. Li et al., 2020).

The isothermal nucleic acid amplification method for detecting nucleic acid targets is void of limitations associated with thermal cycling (Notomi et al., 2000). The isothermal amplification method uses only one temperature and includes helicase‐dependent amplification, recombinase polymerase amplification, and loop‐mediated isothermal amplification (LAMP). All these techniques can be incorporated into a multiplex system during the amplification stage.

The LAMP technique has been proven for COVID‐19 detection (Lamb et al., 2020; Yang et al., 2021). In the LAMP test, DNA polymerase and approximately six primers are used. These primers bind to six different regions on the target genome. Similar to PCR, collected nasopharyngeal or oropharyngeal samples from the patient are added to the tube, followed by DNA amplification and detection via either turbidity, color, or fluorescence. In a recent study by Park et al. (2020), a reverse transcription loop‐mediated isothermal amplification (RT‐LAMP) assay was designed and evaluated. The investigators detected over 100 copies of genomic RNA of SARS‐CoV‐2 using the colorimetric approach. The assays were void of cross‐reactivity with other human coronaviruses. This RT‐LAMP is a promising point‐of‐care test for COVID‐19, but the RNA extraction method needs to be optimized. Compared to PCR, RT‐LAMP requires no centralized laboratory testing or facilities. Hence, it can be conducted with simple instruments (e.g., drying oven or water bath) and have similar sensitivity and specificity (Craw & Balachandran, 2012). The limitations associated with this method include challenges of improving primer and reaction conditions (Udugama et al., 2020). Examples of these testing kits include Real‐Time SARS‐COV‐2 assay and ID NOW COVID‐19 produced by Abbott Diagnostics Scarborough, Inc. SARS‐COV‐2 nucleic acid extraction, isolation, and purification from swabs specimens are required prior to the Abbott assay. The ID NOW assay involves target amplification, heating, mixing, and detection of nucleic acids. The heating step in the ID NOW system could inactivate collected samples (Xie et al., 2020).

In blood‐based serological test, qualitative detection of antibodies (IgM, IgA, and IgG) linked to SARS‐COV‐2 to assess individuals exposed to COVID‐19. The detection of the antibodies via this assay indicates the immune response to SARS‐COV‐2 virus in suspected patients who have been infected previously or with recent COVID‐19 infections. These antibodies can be detected approximately 14 days after infections. Cellex Inc. produces this test kit (Laura, 2020). Before this development, quantitative immunoassay tests such as Dual ELISA were performed. It detects different antibody types (IgA, IgM, and IgG) against SARS‐COV in the blood of suspected individuals. Also, an ELISA kit assay for the detection of SARS‐COV‐2 nucleoprotein has been developed. However, these tests are for research use only and have not been approved by FDA Emergency Use Authorization. Figure 2b shows a time course of COVID‐19 infection and test positivity.

Despite being an accurate, rapid, and straightforward essential tool for elucidation of interactions between several reported cases, antibody‐based detection methods are associated with sensitivity and specificity limitations, and target types are scarce. Moreover, antibody detection may not be suitable for early‐stage infection as the immunoassay could generate negative results since antibodies may still be in the development stage. Another problem could be potential positive results for formerly infected patients. Hence, this calls for massive scrutiny about the accuracy, reliability, and uncertainty of the serological test.

Additionally, a PCR test used for screening COVID‐19 can cost up to $51, while an antibody test costs less than $10 under Medicare (Ellison, 2020). On the frontline of turnaround time, while it takes 15 min to get the serological test result, PCR runs last for about 4–6 h. Due to the back and forth shipment and transfer of samples, results from PCR are procrastinated. As a result, the patient receives results several days after the test run. However, all this has been rectified through the design and invention of the portable point‐of‐care test, and results are received in less than an hour (Table 1).

PCR testing is still highly accurate and embraced by most hospitals, recommended by most doctors for mass screening. This test shows whether a person is still struggling with the virus and can transmit it to others.

According to the FDA, over 5,000,000 test cases are reported weekly via the PCR technique and predict over 1,000,000 difficulties in the coming months for the antibody‐based test.

NGS is a new technique with high accuracy, used to identify and characterize pathogens quickly and result in rapid treatment. The NGS‐based test involves a metagenomic sequencing assay that detects the SARS‐COV‐2 virus with a sensitivity of 500 copies per milliliter (GenomeWeb, 2020). The drawbacks of this technique include high instrument cost, the requirement of bioinformatics experts, difficulty in mass screening, and its test time. It takes at least 2 days to get the test result (Table 1). According to the manufacturers, they are currently focused on the European market. Currently, there are only six approved tests using this technique which is approximately 2% (Figure 2) of the FDA‐approved test with CE marking.

Antigen‐based test or diagnostics is an assay that detects viral protein fragments such as nucleocapsid or spike protein in a specimen collected from nasopharyngeal or nasal swab (Peeling et al., 2021). The viral proteins could be detected via antigen‐capture methods such as antibodies or aptamer (Smithgall et al., 2020). The test can be conducted outside laboratory setting with little training and results can be obtained within 15–20 min. Compared to the molecular test, the antigen testing does not require intensive training, and it is easier, faster, and cheaper to manufacture at a large scale. However, the test is not as sensitive as molecular tests, and cannot detect viral load in specimen. The current FDA‐approved antigen test or kits uses two main approaches (Smithgall et al., 2020): (1) Antigen lateral flow assay with visual read to reflect positivity and (2) immunochromatographic digital assay with instrument read to provide results.

From the limitations mentioned earlier, there is more room to develop improved diagnostic methods. It is critical to continue developing rapid, sensitive, inexpensive, specific, robust point‐of‐care diagnostics for COVID‐19 to enable mass screening exercises, especially in low‐resourced communities. A surveillance diagnostic test that can easily be carried out in different laboratories and outside healthcare settings is urgently needed to prevent the broad and rapid spreading of COVID‐19. It is essential that this test can distinguish between COVs and specifically target the SARS‐COV‐2 virus. This will help individuals with suspected symptoms to rapidly check if they have been infected with the virus.

Despite their promising attributes, only a few tests or assays have been developed using aptamer technology and no commercial product has reached the market yet since the COVID‐19 outbreak (FIND, 2021). Hence, aptamer‐based diagnostic kits can potentially open new doors for rapid and robust detection of SARS‐COV‐2 viruses.

## 4 CONVENTIONAL APTAMERIC NANOSENSOR DEVELOPMENT AS VIRAL DIAGNOSTICS

Aptamers offer a novel approach for targeted diagnosis and treatment of infections. Aptamers have short oligonucleotides that are single‐stranded and consist of either RNA or DNA with the ability to detect a wide range of molecules (Berezovski & Krylov, 2004; Burmeister et al., 2005) including cells, tissues, viruses, and bacteria. They have high specificity and affinity to their target molecules. Aptamers are generated by an artificial method known as Systematic Evolution of Ligands by Exponential Enrichment (SELEX; Zhou et al., 2012). Several SELEX methods including capture‐SELEX, immuno‐SELEX, capillary SELEX, magnetic bead‐based SELEX, high‐affinity resins, have been utilized to detect specific target molecules. In designing nucleic acid sequences specific to target molecules, the SELEX technique employs selecting sequences and replicating them carefully over iterative cycles. Aptamers have between 20 and 90 nucleotides (Wandtke et al., 2015). In generating an aptamer for a target molecule, all feasible aptamers sequence with specific length is selected from a combinatorial library and incubated with the molecule of interest (Kusser, 2000; Somasunderam et al., 2005). During this process, the nucleic acids with high affinity to the target molecule bind to it, and the sequences with low affinity are removed from the pool.

**FIGURE 3:**
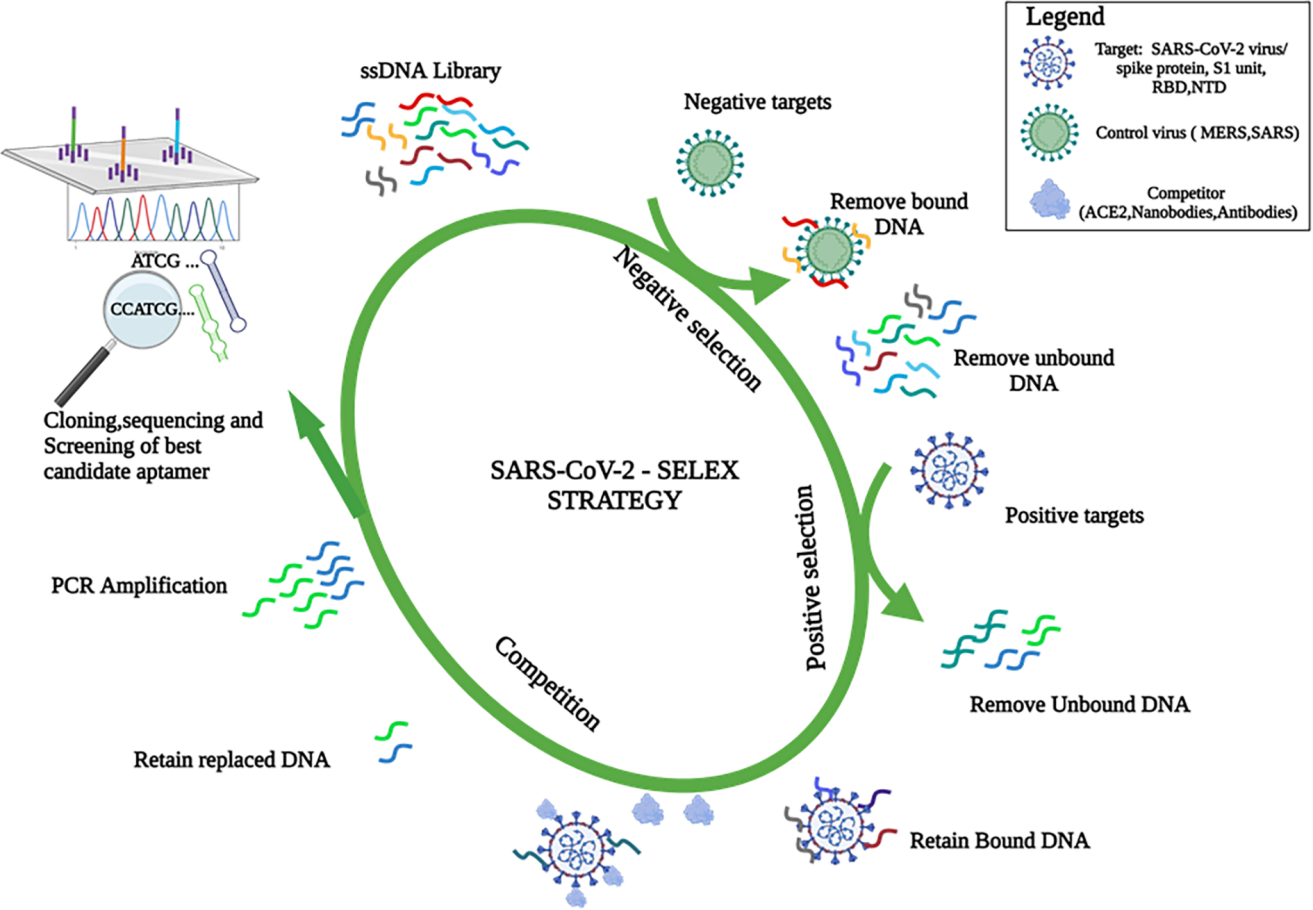
Aptamer development against SARS‐COV‐2 using SELEX process

The high‐affinity nucleic acids and the target molecule complex are recovered and separated. This process is repeated until an aptamer with a low dissociation constant and high specificity towards the molecule of interest is generated. In addition, aptamer obtained from the SELEX method can be integrated with other techniques such as fluorescence resonance energy transfer (FRET; Lee et al., 2017), reverse transcription‐polymerase chain reaction (RT‐PCR; Liu et al., 2020), surface plasmon resonance (SPR; Zou et al., 2019), and capillary electrophoresis (CE; Yang et al., 2021; Zhang et al., 2008) to evaluate binding performance and detection of the aptamer target proteins. This will assist the development of rapid, low‐cost, sensitive, and easy‐to‐use point‐of‐care aptamer based‐biosensors (Samson et al., 2020). Figure 3 shows a robust iterative SELEX process for the development of aptamer against the SARS‐CoV‐2 virus.

Investigating the aptamer binding features under varying conditions of temperature, pH, and conductivity while probing the conformational dynamics of the aptamer presents a useful strategy to optimize the binding performance. Aptamers bind to their targets via unique structural transformations, conformational switching, and signaling, which conventional antibodies cannot exhibit (Oh et al., 2010).

They undergo unique structural changes to generate conformations that facilitate binding to their targets. These conformations, such as G‐quadruplex, enable the aptamer to form stable binding with the target molecule. The free energies associated with the structural transformation can be investigated in silico and experimentally using molecular dynamics simulations and circular dichroism spectroscopy to determine the most stable binding features.

This gives them some advantages over monoclonal antibodies in terms of binding specificity and stability. For instance, they are able to bind to smaller targets or masked relevant binding domains inaccessible by antibodies, and greatly penetrate tissues to elicit higher therapeutic efficacy (Zhou & Rossi, 2017). Antibodies are target or epitope selective and may lose their binding specificity and affinity in the occurrence of any protein modification (Wang et al., 2019).

In addition, the smaller size of aptamers makes them ideal for in vivo use compared to antibodies. The smaller size of aptamers promotes in vivo application due to the ease of membrane penetration, endocytosis, and internalization, ease of formulation into polymeric substrates, and so forth. Also, the smaller size enables easy aptamer formulation into gel and aerosol for intranasal administration or nebulization, straightforward delivery into the respiratory system, and swift neutralization kinetics (Sun, Liu, Wei, et al., 2021). Moreover, immunofluorescence dyes and drugs could be coupled onto aptamers without affecting its functionality and properties (Somasunderam et al., 2005).

**FIGURE 4:**
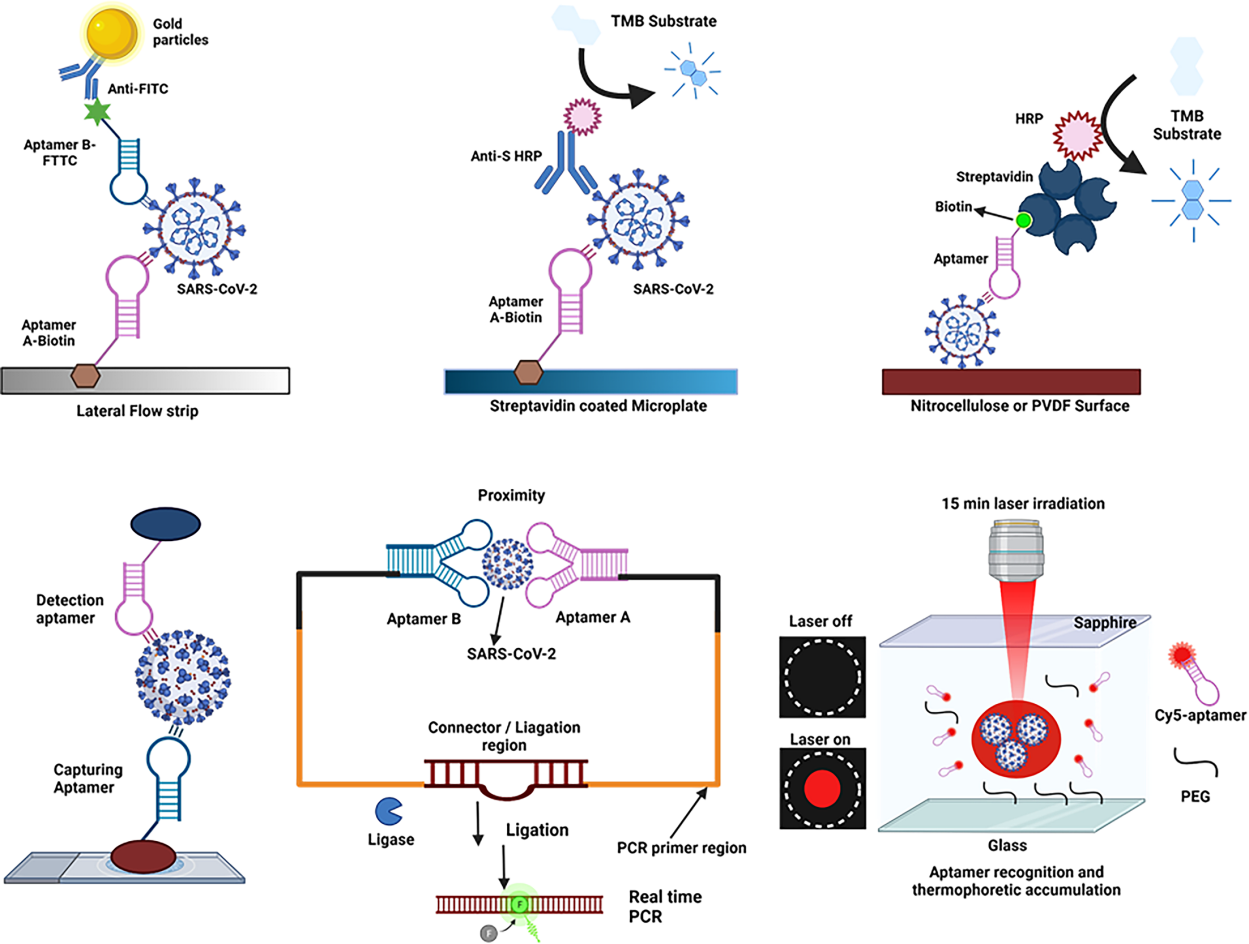
Aptamer‐based biosensing assays/technologies for SARS‐COV‐2 virus detection. (a) Lateral flow device, (b,c) direct aptamer ELISA or enzyme‐linked apta‐sorbent assay (ELASA), (d) sandwich‐type aptamer assay, (e) aptamer assisted proximity ligation assay, and (f) one‐step aptamer‐based thermophoretic assay

Aptamers can be employed in biosensing devices or other technologies as probes, and such biosensors are called aptasensors. These include aptamer‐chemiluminescence immunosorbent assay, aptamer‐based‐surface Plasmon Resonance, aptamer‐based fluorescence, nanoarray aptamer chip assay, proteome microarray, and aptamer‐based Raman spectroscopy (Koteswara Rao, 2021). Figure 4 shows some aptamer‐based diagnostic assays or technologies for rapid, specific, and efficient detection of viral infections. These assays are superior to conventional biosensors that use antibodies as they are stable, have high affinity to targets, are adaptable, and can be developed for a wide range of targets (Mairal et al., 2007), using various transduction mechanisms. Aptasensors have been applied in different forms to detect chemicals, disease biomarkers, and pathogens found in food. Identifying and diagnosing diseases early is crucial to help patients receive initial treatment and better healthcare delivery (Wandtke et al., 2015). In light of this, several antigens and/or viruses have aptamers developed to aid in their diagnosis. Aptamers generated for this purpose target either the whole virus or surface antigens. Over the years, aptamers have been developed for viruses such as hepatitis B and C viruses, human papillomavirus, HIV, Influenza, SARS, Ebola, dengue, herpes simplex virus, West Nile virus (Gopinath, 2006; Gopinath et al., 2016; Gopinath & Kumar, 2013; Gourronc et al., 2013; Moore et al., 2011; Shiratori et al., 2014; Yamamoto & Kumar, 2000; Zou et al., 2019).

## 5 CORONAVIRUS APTAMER SELECTION, CATEGORY, AND ENGINEERING

Using the above‐described selection strategy, various aptamers have been identified and engineered against different parts of the coronavirus structure. In this review, we will classify them into three main categories based on their interaction and/or mechanism of action: spike glycoprotein dependent, Nucleocapsid (N) protein‐dependent (NPD), and helicase or nonstructural protein‐dependent (HPD/NSPD). For the NPD and HPD categories, the aptamers bind, interact, and inhibit the nucleocapsid, NTPase/Helicase protein, or nonstructural protein (NSP), respectively. The spike glycoprotein‐dependent type can be grouped further into receptor‐binding domain (RBD) and nonreceptor binding domain (nRBD) categories. For the RBD group, the aptamers competitively bind to the spike RBD epitope mediating ACE2 engagement, leading to the inhibition of subsequent cell membrane fusion and viral entry and infection. On the other hand, the nRBD aptamers interact with the other part of the spike protein such as the N‐terminal domain to distort its conformational changes leading to viral attachment and entry. Although the nucleocapsid (N) protein and spike glycoprotein RBD categories are the most identified and explored to date, aptamers could be isolated against any target. In the following sections, we review and summarize various aptamers discovered against SARS‐COV viruses. Table 2 shows an overview of aptamers for diagnosis and therapeutic purposes against SARS‐COV‐1 and SARS‐COV‐2.

**TABLE 2:** Some aptamers for diagnosis and therapeutic purpose against SARS‐COV‐1 and SARS‐COV‐2

Category/purpose	Name	Type	No of aptamers	Molecular target	Viral type	SELEX/technology	*K*_D_ (nm)	IC_50,nm_	References
Diagnosis	RNA aptamer‐1; RNA aptamer‐2‐based sensitive probing of N protein	RNA	2	Nucleocapsid N protein	SARS‐COV‐1	Standard SELEX	1.65; 3.35	–	(Ahn et al., 2009)
	DNA aptamer	ssDNA	3	Nucleocapsid N protein	SARS‐COV‐1/SARS‐COV‐2	His‐tagged N protein immobilized on Ni beads	4.93	–	(Z. Chen, Wu, et al., 2020; Cho et al., 2011)
	CoV 2‐ RBD‐1C; CoV2‐RBD‐4C	ssDNA	2	Spike Glycoprotein RBD	SARS‐COV‐2	Ni‐NTA beads SELEX‐ACE2 competition‐Machine learning	5.5; 19.9	–	(Song et al., 2020)
	Np‐A48,Np‐A15,Np‐A58, Np‐A61	DNA	4	Nucleocapsid N protein	SARS‐COV‐2	Standard SELEX	<5	–	(Zhang et al., 2020)
	Apt‐PLA; N48,MN48‐1, N58,MN58‐1 Apt‐S‐79s, Apt‐S‐268s	DNA	6	Nucleocapsid N protein, spike protein, ACE2	SARS‐COV‐2	Standard SELEX	–	1790–1830	(Liu et al., 2020)
	SNAP 1,3 1.5, 1.66	DNA	4	N‐terminal domain	SARS‐COV‐2	Standard SELEX	<80	–	(Kacherovsky et al., 2021)
	NSP10‐053, NSP10‐001, NSP10‐010	RNA	3	Non‐structural protein, NSP 10	SARS‐COV‐2	Computation, protein docking, MD simulation	–	–	(Kothandan et al., 2021)
Therapy	NG series	ssDNA	5	NTPase/Helicase	SARS‐CoV‐1	Ni‐NTA magnetic beads SELEX	5.4–26.8	17.5–120.8	(Shum & Tanner, 2008)
	ES15	RNA	5	NTPase/Helicase (NSP10)	SARS‐CoV‐1	Standard SELEX	–	1.2 (helicase) 77 (NTPase)	(Jang et al., 2008)
	SP 1–7, SP6 variants	ssDNA	18	Spike protein S, Non‐RBD	SARS‐CoV‐2	Automotive Selection/His‐tagged Spike protein on immobilized on Ni‐NTA Magnetic beads	<20	–	(Schmitz et al., 2021b)
	nCoV‐S1‐Apts 1–6	DNA	6	Spike protein S1 RBD	SARS‐CoV‐2	Capillary electrophoresis (CE)‐based SELEX	0.118–85	80.12	(Yang et al., 2021)
	RBD‐PB6, RBD‐PB6‐Ta	Monomer DNA	2	Spike protein S1 RBD	SARS‐COV‐2, wild‐type, and variant (alpha and beta)	SELEX	0.4	500–1500	(Valero et al., 2021)
	Multimeric aptamer, RBD‐PB6‐Ta dimer, RBD‐PB6‐Ta trimer	Dimer, Trimer	2	Spike Protein S1 RBD	SARS‐COV‐2, wild‐type, and variants (alpha and beta)	SELEX and Multimerization	72^a^/39^a^	387/46	(Valero et al., 2021)
	CoV2‐1‐8 bivalent (cb‐CoV2‐6C3)	DNA	10	Spike protein RBD	SARS‐COV‐2	RBD Ni‐beads SELEX ACE2‐competition MD simulation	0.13	0.42	(Sun, Liu, Wei, et al., 2021)
	Aptamer cocktail, CoV2‐1C, CoV2‐4C, and CoV2‐6C3	DNA	1	Spike protein RBD	SARS‐COV‐2, Wild‐type, and variant	SELEX and aptamer mixture	–	–	(Sun, Liu, Song, et al., 2021)
	Spherical Multivalent aptamer, SNAP	DNA conjugate to Gold nanoparticle	1	Spike protein RBD	SARS‐COV‐2, Wild‐type, and variant	SELEX and gold conjugation	3.90^a^	142.8^b^	(Sun, Liu, Song, et al., 2021)

*Note*: Diagnostic aptamers have *K*_D_ (nm) values, and therapeutic aptamers have inhibition concentration values (IC_50_).

Abbreviation: RBD, receptor‐binding domain.

^a^ Unit of *K*_D_ or IC_50_ is in picomolar (pM).

^b^ Unit of *K*_D_ or IC_50_ in femtomolar (fM).

### 5.1 Aptamers for SARS COV‐1 theranostic application

Various diagnostic aptamers for SARS‐COV have been discovered recently. An aptamer with a dissociation constant of 4.93 nM has been generated to target the nucleocapsid protein of SARS COV (Cho et al., 2011). From the ELISA analysis, this ssDNA aptamer is bound explicitly to the nucleocapsid protein. In comparison with antibodies, the authors further showed that the ssDNA aptamer could detect the SARS‐COV nucleocapsid protein efficiently via Western blot analysis. In an earlier development by Ahn et al., an RNA aptamer was isolated or selected to target the nucleocapsid protein, and a 1.65 nM dissociation constant was obtained (Ahn et al., 2009). Fluorescence imaging showed a detection limit of 2 pg/ml. The aptamer had a better binding against N protein than the antibody tested (*K*_D_ = 2.1 μM).

Furthermore, Ahn et al. immobilized RNA aptamers onto a streptavidin‐coated plate to detect and capture N protein. The signal transduction was achieved using polyclonal anti‐N antibody and FITC‐labeled anti‐rabbit IgG secondary antibody. The system reached a detection limit of 20 pg/ml for the N protein, reminiscent of ELISA using monoclonal antibodies (He et al., 2005). Also, some therapeutic aptamers against SARS‐COV have been discovered. In a study by Jang et al. (2008), RNA aptamers were generated for the NSP10 (NTPase/Helicase) found in SARS coronavirus. They reported that the isolated RNAs efficiently bound and inhibited duplex DNA unwinding activity of SARS‐COV helicase by approximately 85%. The IC_50_ value was 1.2 nM. However, they stimulated infinitesimal change on ATPase activity of the helicase protein in the presence of a cofactor.

Similarly, Shum and colleagues reported the binding between an immobilized DNA aptamer on Ni‐NTA magnetic beads and SARS‐coronavirus helicase (Shum & Tanner, 2008). Using ATPase and fluorescence resonance energy transfer (FRET) based assay, all the selected/isolated aptamers demonstrated inhibitory activity against the SARS‐COV helicase with low apparent *K*_m_ values. The SARS‐COV helicase can unwind its duplex nucleic acid (DNA and RNA) to enable the replication and proliferation of the virus (Ivanov et al., 2004; Tanner et al., 2003). Through circular dichroism and gel electrophoresis techniques, the investigators identified two different aptamer classes, namely, G‐quadruplex and non‐G‐quadruplex. Interestingly, non‐G‐quadruplex aptamer cloned efficiently and specifically inhibited the SARS‐COV helicase‐unwinding activity with IC_50_ ranging from 17.5 to 120.8 nM. The opposite was observed for the G‐quadruplex aptamer class.

Also, Roh and Jo (2011), specifically detected SARS‐COV nucleocapsid protein in a one‐spot experiment (Roh & Jo, 2011). Using an immobilized SARS‐COV N protein‐glass chip system, the investigators achieved this with RNA aptamer conjugated to quantum dots (QDs). Fluorescence imaging showed a detection limit of 0.1 pg/ml.

Recently, researchers have developed new creative diagnostic kits using a pioneering imaging technology called Mango for its vivid color to sensitively detect RNA molecules, helping to improve the screening of viruses such as coronavirus while enabling fundamental discoveries into the functioning of cells (Buquliskis, 2020). The Mango system was made up of an RNA aptamer which acts like a magnet targeting the dye molecules. The dye becomes excited when bound and glows brightly. They concluded that the Mango NABSA kits could be used to detect pathogens such as the positive‐stranded RNA coronavirus faster and more efficiently.

### 5.2 Aptameric theranostics for SARS‐COV‐2 coronavirus: Recent development

The search for point‐of‐care tools for rapid detection of the SARS‐COV‐2 is in the utmost need. At the York‐based biotechnology company Aptamer group, the research team has recently isolated a group of monoclonal aptamers with binding specificity for SARS‐COV‐2 spike protein (Aptamer Group, 2020). It has been reported that the aptamer set shows significantly stronger interactions with the spike protein of the novel coronavirus. Also, other cross‐reactive aptamers which bind to the spike protein of other β‐coronaviruses (SARS, MERS) were discovered. Also, the Aptamer group is developing a point‐of‐care test for rapid detections of SARS‐CoV‐2 (Farewell, 2020). This test is intended to generate results within 141 s. Also, Rapid microbiology has employed a similar approach to develop Pinpoint's low‐cost handheld aptameric nanosensor‐based diagnostic assay/device to detect and test COVID‐19 accurately within 30 s (Microbiology, 2021).

Interestingly, this test does not require clinicians or experts and can differentiate the SARS‐COV‐2 virus from influenza and other recurrent respiratory pathogens. In addition, Sharma and co‐workers (Prasad, 2021) from Faridabad‐based Translational Health Science and Technology Institution (THSTI) developed aptamer‐based immobilized immunosorbent assay for detection of SARS‐COV‐2 virus using two DNA aptamers. These aptamers bind to the spike protein and detect coronavirus with 90% selectivity and 97% specificity.

After five selection rounds, Zhang et al. (2020)reported four DNA aptamers targeting the SARS‐COV‐2 nucleocapsid protein (Np). These aptamers displayed super binding affinity (<5 nM) and could bind to Np in a sandwich‐type format. Incorporating these sandwich‐type aptamers into ELISA and gold nanoparticle immunochromatographic strips, the authors detected Np at a LOD of 1 ng/ml, buttressing their application in future clinical samples. By employing two of these aptamers (N48, N58), Liu and colleagues further developed an aptamer‐assisted proximity ligation assay as a generic method/platform (Apt‐PLA) for accurate, ultrasensitive, and specific detection of serum nucleocapsid protein, measurement of ACE2–spike protein interactions, investigation and screening of other neutralizing aptamers (Liu et al., 2020). This system utilizes two aptamers to bind the same protein target, ligate DNA region closely, and initiate ligation‐dependent qPCR amplification within 2 h (Figure 4e). The authors showed that the Apt–PLA system showed a significantly low threshold value (Ct) for qPCR curves, high ligation efficiency for the N protein, and vice versa for control samples, including other competing proteins. Compared to the SARS‐COV‐2 N protein at a 2 ng/ml concentration, no significant change was observed for competing proteins used in this study, suggesting highly sustained selectivity of these two aptamers. In addition, the Apt‐PLA limit of detection (LOD) is on parity with the commercial ELISA kit (37.5 pg/ml vs. 50 pg/ml), but much better than that of new developed half‐recent strip lateral flow assay (LOD = 0.65 ng/ml). This system possesses the remarkable advantage of short operation time and is on par with commercial ELISA kits in detecting SARS‐COV‐2 protein in human serum. According to the authors, the Apt‐PLA system can be adapted as an alternative generic method to gold standard neutralization tests to screen potential neutralizing antibodies or DNAs due to its shorter time‐to‐results (2 h). To achieve this, they incorporated three different aptamers (Apt‐S‐79s, Apt‐S‐268s, random DNA), Spike S1, and ACE2 proteins into their system. Compared to the random DNA, Spike S1 targeting aptamers Apt‐S‐79s and Apt‐S‐268s demonstrated a significant dose‐dependent neutralization effect with respective IC_50_ of values 1.83 and 1.79 μM. Also, ACE2 protein displayed a strong dose‐dependent neutralization potency against Spike S1 with an IC_50_ value of 284.5 pM.

In another research, Song and co‐workers recently isolated and selected aptamers that detect or bind to the S protein's receptor‐binding domain (RBD; Song et al., 2020). The authors used artificial intelligence and machine learning techniques to discover two DNA aptamers with 5.8 and 19.9 nM binding affinities. The aptamer‐SARS‐COV‐2 S RBD interactions were monitored using His‐tag modified Ni‐beads model/system, modeling, and flow cytometry. Both aptamers partially block and occupy the ACE2 binding epitope on the SARS‐COV‐2 RBD. In a tandem study, the same authors identified lead aptamer CoV2‐6 that form bridge interaction with RBD to block ACE2 engagement. Using molecular dynamics (MD) simulation, it was revealed that CoV2‐6 sterically covers and/or embeds in the ACE2 binding interface on the RBD, implicating its competition with ACE2 for the same epitope. After removing unnecessary nucleotides, truncated aptamer, CoV2‐6C3 had a higher binding affinity (44.78 nM vs. 84.64 nM) and did not bind other viral pathogens RBD or spike protein, indicating its specificity and selectivity. The authors further engineered this truncated variant into a circular bivalent form (cb‐CoV2‐6C3). cb‐CoV2‐6C3 had better and/or improved stability and binding affinity over the monovalent aptamer after subjecting unfavorably harsh conditions such as human plasma (95%) and cell media (containing 10% FBS). Intriguingly, circular aptamer had a 344‐fold higher RBD binding affinity than the monovalent counterpart (0.13 vs. 44.78 nM). Furthermore, circular aptamer displayed a high neutralization profile and strongly blocked both pseudovirus and authentic virus infections with respective IC_50_ values 9.68 and 0.42 nM, which is on parity with other reported neutralizing antibodies.

In another study, Z. Chen, Wu, et al. (2020) investigated and modified a previously reported aptamer for SARS‐COV N protein by Cho et al. (2011) to detect SARS‐COV‐2 N protein due to the similarity between the N protein of the two coronaviruses. The authors tested all three biotinylated aptamers using an enzyme‐linked aptamer binding assay (ELAA). All three aptamers are bound to the SARS‐COV‐2 N protein with detection as low as 10 ng/ml. Intriguingly, aptamer 2 and 3 detected N protein in human serum.

Tan and colleagues (Deng et al., 2021) have developed a rapid one‐step aptamer‐based thermophoretic assay that can detect viral particles or pathogens directly and/or accurately. By deploying integrated SARS‐COV‐2 spike protein cy5‐conjugated aptamers and polyethylene glycol (PEG)‐enhanced thermophoretic accumulation, this assay detected both pseudotyped SARS‐COV‐2 virus and infected oropharyngeal swab within 15 min at LOD of ∼170 particles/μl^−1^ without requiring any pretreatment.

Recently, Combining SELEX, biolayer interferometry (BLI), and cryogenic electron microscopy (Cryo‐EM), Kacherovsky et al. (2021) discovered the first novel DNA aptamers with subnanomolar binding to the N‐terminal domain of the SARS‐CoV‐2 spike glycoprotein including spike protein variants (S, S2P, and S‐B.1.1.7), but do not interfere with ACE2‐spike S1 binding interface. Interestingly, these aptamers did not bind to both SARS‐COV‐1 and MERS‐CoV Spike S1 and NTD, corroborating their specificity. The investigators further applied its lead candidate (SNAP1) in lateral flow assay and aptamer‐antibody sandwich ELISA (Figure 4a,b) to detect Spike protein and UV‐inactivated SARS‐COV‐2, implicating aptamer versatility and application in other diagnostic assays. To their surprise, SNAP1, in its truncated formats (SNAP 1.5, 1.66), retained their binding affinities to SARS‐COV‐2 S1 and NTD. All these discoveries open room for the development of point‐of‐care diagnostics to detect the COVID‐19 and SARS‐CoV‐2.

On the frontier of therapeutics, Mayer and co‐investigators (Schmitz et al., 2021a) have identified an aptamer, SP6, with specific interaction and high affinity to SARS‐COV‐2 spike protein (21 and 13 nM) under different conditions (37 and 25°C) but do not bind with RBD nor block the spike protein ACE2 engagement. SP6 did not bind to isolated RBD, ACE2 protein, or to SARS‐COV‐1 spike protein. Compared to the unmodified state, 5′ modification with biotin, Cy5, and hydroxyl did not significantly affect aptamers—spike protein binding properties. Interestingly, although point‐mutated variants of minimal aptamer variant SP6.34 (from parental SP6 aptamer) had abolished spike protein binding, the truncated versions had improved or comparable binding to SP6 aptamer. Hence, a small aptamer size would merit synthesis and drug delivery. Moreover, the authors showed that SP6 aptamer neutralized pseudotyped SARS‐COV‐2 virus and virus bound cells in a dose‐dependent manner via RBD independent targeting routes, demonstrating this aptamer as a promising inhibitor that could rival escape variants that reduce the potency of RBD‐targeted antibodies.

Also, Huang and colleagues (Yang et al., 2021) reported six new highly potent DNA aptamers that blocked and neutralized the SARS‐COV‐2 virus. These aptamers displayed high specificity and nanomolar affinity for spike S1 protein (*K*_D_ < 85 nM) and blocked RBD‐ACE2 engagement. Interestingly, lead aptamer nCoV‐S1‐Apt1 detected S1 protein in human serum and reduced/inhibited SARS‐COV‐2 infections in pseudovirus model with IC_50_ of 80.12 nM, implicating its potential as an ultrapotent neutralizer against SARS‐COV‐2 virus.

The above‐reported aptamers exhibited single site viral blocking and had unsatisfactory efficacy (both *K*_D_ and IC_50_ at the nanomolar range). It was also difficult to suppress SARS‐COV‐2 mutational escape and infections. Multimerization of certain SARS‐COV‐2 biologics such as host receptor ACE2, nanobodies, and single‐chain variable fragments (scFv) into avidity therapeutics have been embraced recently as an effective treatment approach to curb recurrent viral mutation leading to escape issues (Cuesta et al., 2010; Jing & Procko, 2021; Obeng et al., 2022). Therefore, similarly, rational mixtures or cocktails and aptamer multimerization strategies would strongly enhance their binding efficiency (from nanomolar to picomolar range) and improve SARS‐COV‐2 neutralization potency to resist potential viral escape mutation development. For instance, Sun, Liu, Song, et al. (2021) demonstrated that aptamer cocktails displayed the higher RBD and pseudovirus neutralizing efficiency compared to their individual aptamer counterparts with the same concentration. In terms of aptamer multimerization, Valero et al. (2021) engineered dimeric and trimeric versions of RBD‐PB6‐Ta aptamers via a scaffolding or poly(A) linkage approach. Compared to the monomeric aptamer, both dimeric and trimeric forms demonstrated an enhanced spike protein binding avidity (*K*_D_ values of 0.4 nM, 72 pM, and 39 pM for monomer, dimer, and trimer, respectively) and efficiently neutralized real SARS‐COV‐2 virus with respective IC_50_ values of 1.5 μM, 387 nM, and 46 nM.

## 6 NANOTECHNOLOGY ADVANCES ON CORONAVIRUS APTAMER THERANOSTIC AND VACCINES

**FIGURE 5:**
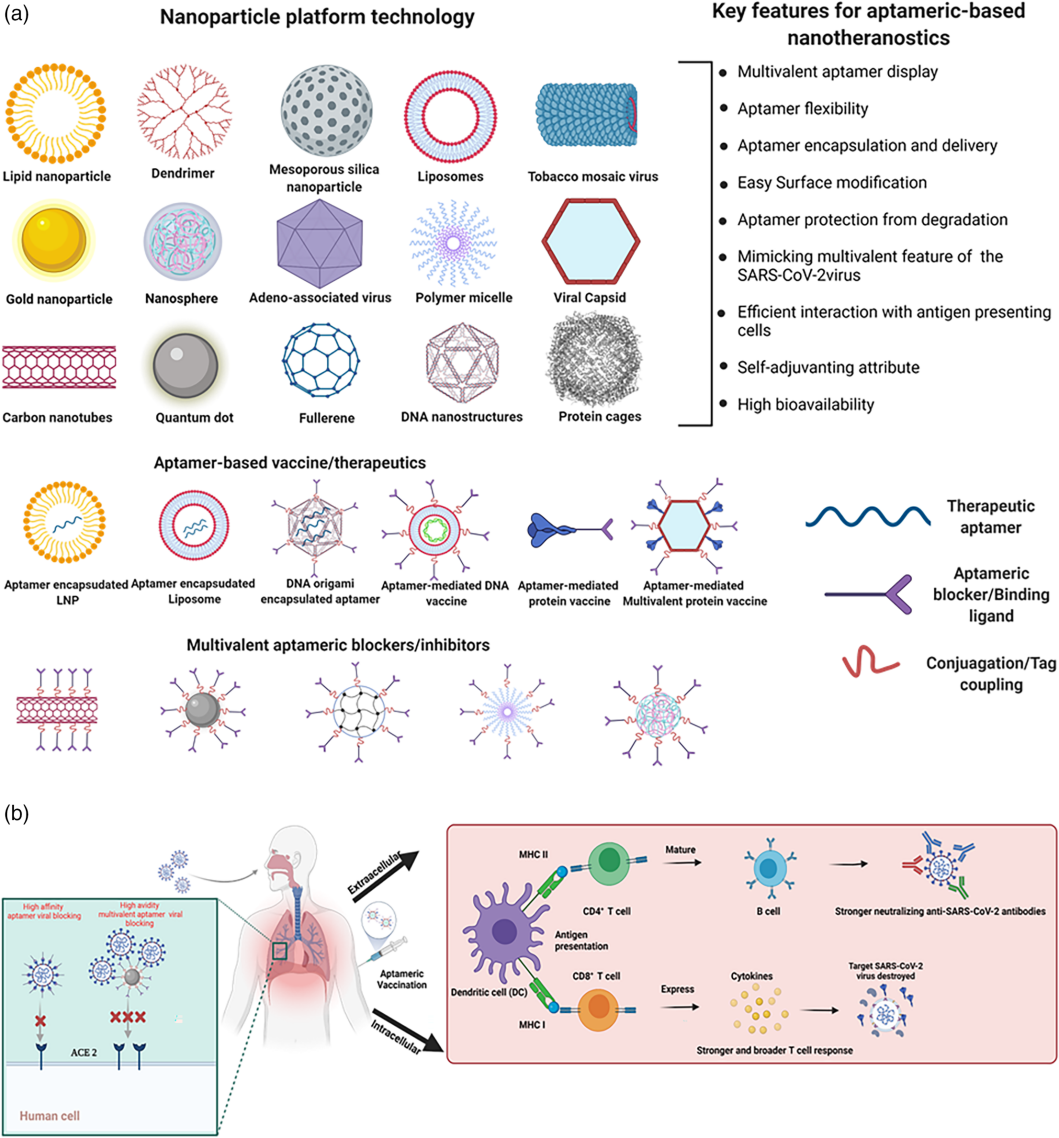
Proposed nanotechnology approaches and advances in nanotherapeutic aptamer. (a) Some examples of biocompatible nanoparticle platform for the development of coronavirus aptameric therapeutics, (b) engineered aptameric‐based blockers, therapeutics and vaccines, and (c) mechanism of Aptameric‐mediated SARS‐COV‐2 inhibition, treatment, and immunity development after aptamer‐mediated vaccination. Multivalent aptameric blocker demonstrates high avidity blocking compared to monomeric molecules. The Aptamer‐directed antigen is processed by the dendritic cell, and MHC‐I and MHC‐II present epitopes for effective production of CD8^+^ cytotoxic T cells or CD4^+^ T helper cells required for anti‐SARS‐COV‐2 antibody production (or a combination thereof)

Despite the tremendous efforts and countermeasures in development against the SARS‐COV‐2, there still prevail frequent circulating mutations and viral variants across the globe leading to immune escape (Harvey et al., 2021). Although none of these has been reported of described aptamers to date, advanced approaches are required to prevent future escape. The SARS‐COV‐2 virus enters the host cell via stable multivalent binding events of the ligands (spike glycoprotein) and the ACE2 receptors despites its low binding affinity of single spike protein binding sites to ACE2 receptor. We propose that synthetic multivalent entry aptameric blockers mimicking and/or building upon these spontaneous occurring multivalent binding phenomena may offer a striking effect. This may increase the longevity, sensitivity, breadth, and potency of the aptamers against the coronavirus and its related future mutational variability. For instance, some effective influenza and Dengue virus inhibitors displaying multiple ligands such as sialic acids, aptamers, and antibody‐derived peptides have been designed to tandemly bind to many envelope protein domains III and densely packed hemagglutinin epitopes, respectively (Kwon et al., 2020; Lauster et al., 2017). In addition, low aptamer threshold would be required to prevent or reduce the virus infection in the lower and upper respiratory airways. To achieve this, medically relevant customizable molecular scaffolds (Knappe et al., 2021; Kwon et al., 2020; Veneziano et al., 2020) and biocompatible particulate nanotechnology platforms (Lauster et al., 2017, 2020; Ogata et al., 2016; Ueda et al., 2020) with tremendous promises can be utilized to incorporate both sensing, inhibiting and neutralizing aptamers to address above‐mentioned challenges on both prophylactics and treatment fronts (Figure 5a). These include liposomes, viral‐like particles, polymeric nanoparticles/micelles, dendrimers, protein cages, lipid nanoparticles, quantum dots, and DNA nanostructures. The surface area of these ligand scaffolds and nano‐platforms is large to incorporate these aptamers at optimal concentration, densities, and/or valency for emulating three‐dimensional (3D) geometric arrangement, spatial distribution, and orientation of the spike (S) protein (Huang et al., 2020; Ke et al., 2020), two‐dimensional (2D) spatial pattern matching of the virus (Kwon et al., 2020; Ueda et al., 2020), selective enhanced/multivalent binding and avidity (Curk et al., 2017; Papp et al., 2011; Figure 5b). These spatially matched viral patterns, interactions and occupancy of larger ACE2 binding interfaces enable suitable steric hindrance and potential disruption of the trimeric spike protein conformational transition from prefusion to postfusion state, hindering viral infection and pathogenicity (Benton et al., 2020). In addition, these platforms possess superb bioavailability, longer blood circulatory properties, and pharmacokinetics profile (Gill et al., 2007; Li et al., 2018), which makes them perfect for curbing lung‐related or respiratory diseases of which COVID‐19 is no exception. Due to their small size, aptamers are rapidly cleared through renal filtration, which poses challenges for their in vivo use (Kovacevic et al., 2018). Therefore, these nanotechnology platforms and strategy will increase their molecular size to minimize renal elimination and protect the aptamers from nuclease degradation (Cutler et al., 2012; Mirkin et al., 2019).

In the past decades, several remarkable chemistries, functionalization, and conjugation approaches have been utilized to incorporate some biomolecules onto the above‐mentioned scaffolds and nanoplatforms. Similar approaches can be used in tandem to formulate coronavirus aptamers into their multivalent forms to enable desired enhanced binding and viral neutralization. However, it is required that these strategies are site‐specific and biorthogonal to obtain the optimal number of aptamers on the molecular scaffolds or nanoparticles. These promising strategies include biotin‐streptavidin/avidin ligation (Xu & Wegner, 2020), Sortase‐mediated ligation (Chen et al., 2015), Click chemistry or a combination thereof (Sortase‐Mediated Click‐Chemistry Functionalization; Moliner‐Morro et al., 2020), and peptide–peptide ligation (Andersson et al., 2019; Keeble & Howarth, 2020; SpyCatcher/SpyTag and snooptag/snoopcatcher) for multimeric display of aptamer.

Recently, Sun, Liu, Song, et al. (2021) developed a spherical neutralizing aptamer‐gold nanoparticle strategy for synergistic blocking of ACE2–RBD interaction, multivalent multisite spike binding, broad, and potent virus neutralization. Compared to the monovalent aptamers and their respective cocktails, the multivalent aptamer‐gold conjugates display exceptional binding avidity against the spike RBD (*K*_D_ = 3.90 pM) and potent actual SARS‐COV‐2 virus neutralization (IC_50_ = 142.80 fM), which is 2–3 order magnitude lower than reported neutralizing aptamers and antibodies. It further blocked and suppressed three mutant pseudovirus infections.

Currently approved vaccine candidates for SARS‐COV‐2 employ multiple vaccine platforms and these include recombinant spike protein and/or subunits (S1, S2, and RBD), DNA/RNA‐based vaccines, lipid encapsulated mRNA formulations, live virus attenuated vaccines, RBD/spike protein displayed‐virus‐like particles, replication‐competent/incompetent viral vectored vaccines, and inactivated virions (Krammer, 2020). However, none of these vaccines directly or precisely target professional antigen‐presenting cells (APC). Moreso, these vaccines demand specialized storage conditions. Therefore, vaccines with targeted antigen delivery and better stability would offer better hopes to curtail coronavirus transmission and mortality. To this end, we propose a strategy where aptamers that recognize professional class I or II Major Histocompatibility Complex (MHC) APCs are fused/conjugated to either coronavirus spike protein/conserved subunits or on viral capsids as antigen complex vaccines (Figure 5b,c). These vaccines would fast‐track antigens access to processing and presentation pathways to enhance or stimulate robust humoral (binding and neutralizing antibodies) and cellular immunity against coronavirus (Figure 5c). Due to their higher tissue penetration ability, direct, and specific APC targeting, aptamer within the antigen‐complex vaccines will reduce the continuous laborious migratory and sampling burdens on the APC such as dendritic cells to locate antigens (spike protein or receptor‐binding domain or other conserved subunits). Hence, speeding up the engagement process between dendritic cells and T cell in the lymph nodes (Curato et al., 2019). Similar efforts have been demonstrated by Ploegh and colleagues using nanobodies to recognize class II MHC and present antigens to professional APCs (Pishesha et al., 2021; Woodham et al., 2018). However, compared to aptamers, nanobodies are more susceptible to potential immunological responses and problems (Van Bockstaele et al., 2009; Vincke et al., 2009). Also, aptamers can be delivered as promising vaccines adjuvants or immunopotentiators (Pastor et al., 2013).

In summary, despite the promise of these multivalent aptamer prophylactics, therapeutics, and vaccines, safety assessments and validations of the final products are warranted.

## 7 FUTURE PERSPECTIVE

The development of rapid, cheaper, specific, sensitive, and user‐friendly alternative antagonists/ligands/tools for CoVs structural proteins would contribute to the effective diagnosis and treatment of coronaviruses. New approaches have been developed to enhance SARS‐COV‐2 detection but have not been approved by the FDA yet. Therefore, a practical approach is via a combination of the methods above to avoid the limitations of a single test and methods.

Due to their biophysical characteristics or potentials, aptamers have previously been demonstrated and hailed as bioprobe/affinity ligands for rapid sensing, screening, and detection of infectious or pandemic pathogens and virulent diseases. These characteristics make them ideal candidates for designing and developing point‐of‐care tests (POCT) to diagnose and combat COVID‐19 and other future pandemics. Briefly, in comparison to molecular‐based assays or diagnostic technologies, aptameric sensors have advantages such as (i) ease of generation for several targets (including toxins and non‐nucleic acid targets; Radom et al., 2013); (ii) non‐requirement of sample preparation (Wark et al., 2010); (iii) the capacity to develop biosensing technologies that are simple, rapid, less expensive, and can be used in real‐time (Garibyan & Avashia, 2013).

Additionally, although it is subjected to application format, aptameric sensing technologies can be designed to depend on labels, centralized, or clinical laboratories, and well‐trained staffs. Hence, it can be developed for detection or testing for COVID‐19 cases at home, and this will reduce traveling to clinical/healthcare facilities which could contribute to rapid diseases transmission and escalation. Similar to molecular diagnostics, aptasensors are highly specific, sensitive, and can be applied or incorporated into multiplex systems or applications (de‐los‐Santos‐Álvarez et al., 2008; Yang & Rothman, 2004).

Through aptamer immobilization on two‐dimensional (Glass slides, silicon, chips, and polymethylmethacrylate) and three‐dimensional (Microspheres made of magnets, agarose, silica, monoliths, and polystyrene) support, many aptamer sensors with improved characteristics or properties against coronaviruses can be developed for dual functions, detection, and screening (Danquah & Forde, 2008; Deng et al., 2012; Madru et al., 2011). The 3D immobilized aptasensors are highly efficient and possess a higher surface‐area‐to‐volume ratio (Acquah et al., 2015). Also, they have high loading capacity, are less prone to steric hindrance, and can be used efficiently in multiplex assays. Moreover, 3D immobilized aptasensors have demonstrated high throughput ability and are cost‐effective. They can be used to detect and capture pathogens into the support matrix. The development of 3D aptasensors assays are quicker, hence, a criterion for rapid, specific, and sensitive screening of pandemic pathogens.

These improved aptasensors technologies will be essential for effective mass screening during pandemics and possibly for detecting asymptomatic cases and low pathogen limits in suspected individuals since it takes at least 2–14 days for suspected patients to develop symptoms of COVID‐19 infections (Gao et al., 2021).

Recently, great strides and milestones have been reached on aptamer discovery through integrated artificial intelligence‐based technologies (Song et al., 2019), multi‐omics, high throughput screening, and in silico, or computational methods (Ahirwar et al., 2016; Akbaripour‐Elahabad et al., 2016; Cataldo et al., 2018; Hamada, 2018). These have come to solve problems of efficient and successful identification of new or high‐performance aptamers from the combinatorial library using the SELEX technology. Additionally, high cost, labor, and time further limit the SELEX process (Emami et al., 2020). These computational methods have revolutionized the separation and identification efficiencies; decreased amplification bias and nonspecific binding; and enabled the expansion of different base types. Several machine learning methods have emerged for the identification of high‐performance aptamers. These include sequence clustering‐based methods (Alam et al., 2015; Hoinka et al., 2014) and motif finding‐based methods (Hiller et al., 2006; Hoinka et al., 2014). Unfortunately, the former had limitations such as high amplification bias, and the binding process is not specific. Although developed to over the challenges of the former, the later studies face limitations such as high computational and analysis costs. Also, the general secondary structures of aptamers are not considered, which makes it absolutely choice dependent.

Both methods were unable to identify highly efficient aptamers from the SELEX pool. Some of these challenges have been addressed in different studies. In recent work using machine learning‐based classification, Song et al. (2019) developed an Algorithm (SMART‐Aptamer) to select aptamers from the sequencing data SELEX pool to address some of the challenges above. The investigators rapidly identified three aptamers with excellent affinity, selectivity, and accuracy for epithelial stem cells, human embryonic cells, and blood cells. The aptamer (SJ‐3C2) demonstrated a strong binding ability with a *K*_D_ value in the nanomolar range of 41.43 ± 1.84 nM. In a recent study, using the same technology and ACE2 competition‐based aptamer selection method, they isolated two novel aptamers (COV2‐RBD‐1C and CoV2‐RBD‐4C) against SARS‐CoV‐2. Both aptamers interacted and bound to the RBD Ni‐NTA beads model with *K*_D_ values in the nanomolar range (5.8 and 19.9 nM).

Using large‐scale Molecular Dynamic simulation, Cleri et al. (2020) tested the ability of these aptamers to recognize, interact and bind to different subdomains of SARS‐CoV‐2 spike protein S1, blocking virus‐ACE2 receptor binding.

In addition, Kothandan et al. (2021) designed RNA aptamers that bound strongly with the nonstructural protein using combined computational, protein docking, and molecular dynamic simulation approaches.

**FIGURE 6:**
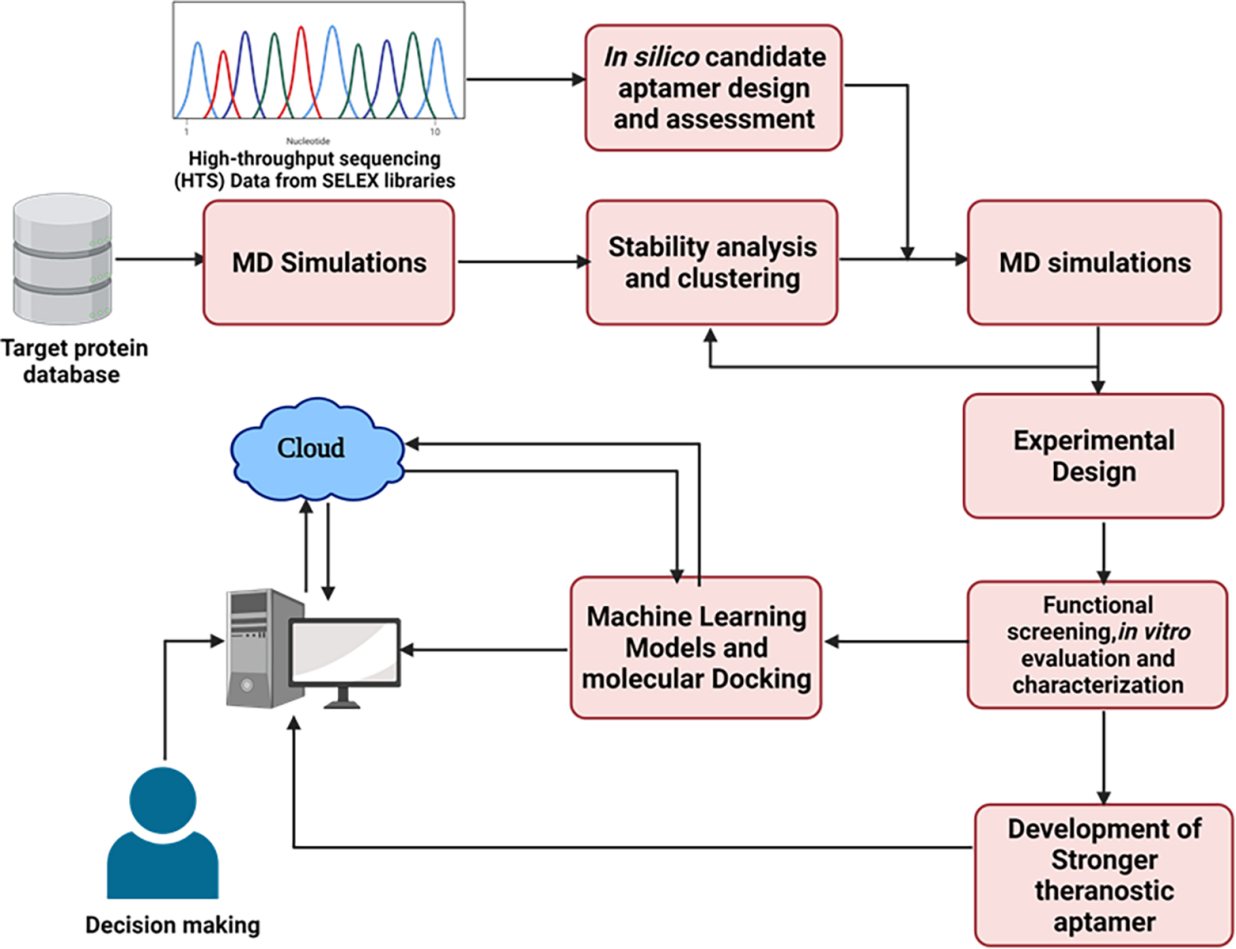
A proposed computational approach for the discovery and development of ultrasensitive aptamer for SARS‐COV‐2 theranostic. This consists of MD simulation, clustering via machine learning, and molecular docking approach. Reprinted and adapted from Sabbih et al. (2021). Open Access^©^ 2021 by Sabbih et al. (2021). Biotechnology Progress

Thus, combining conventional SELEX experimental methods with in silico approaches such as molecular dynamics (MD) simulation and machine learning, improved/enhanced aptamer sensing technologies, and therapeutics can be developed to target different structural proteins/epitopes of SARS‐COV‐2. Figure 6 shows a proposed in silico pipeline to optimize and develop ultrasensitive and highly specific aptamers against different SARS‐COV‐2 structural proteins. This critical review highlights the capability of high‐affinity aptamers as a promising tool for biosensing and treatment of COVID‐19. The juxtaposition of aptamer‐based, nucleic acid‐based, and serology or immuno‐based assays have been summarized in Table 3. Despite the potential merits of aptamer over antibodies, serological assays, and nucleic‐based amplification techniques for diagnostic and therapeutic applications, some significant issues or challenges need to be addressed to realize the full‐scale development and application of aptasensors. First, there could be a low diversity of aptamer due to the low chemical variety of native RNA or DNA molecules. This problem has been solved via the use of modified DNA or RNA molecules.

**TABLE 3:** Comparison of aptamer‐based, nucleic acid‐based, serological‐based, and antigen‐based assays

Parameter	Aptamer based assays	Nucleic acid‐based assays	Serological assays	Antigen‐based assay
Selection	In vivo	In vitro	In vitro	In vitro
Production	Chemical solid‐phase synthesis and machine learning techniques (Easy production)	A viable organism is not required	Immune response in animal origin	Easy production
Sample preparation	Not necessary	Pre‐analysis and additional detection after amplification is required	Pre‐analysis and preparation required	Not necessary
Cost	Cost‐effective	Expensive	Expensive	Cheaper
Specificity	Highest	Higher: Effective at low viral loads	Lower	Moderate: cannot detect low viral load
Sensitivity	Highest	Higher	High	Moderate
Target	Wide range of targets	Limited targets to nucleic acid	Limited to immunogenic targets	Viral proteins, N and S
Compatibility	Compatible with different diagnostic techniques	High susceptible to contamination	–	Less susceptible to contamination, compatible with
Rapidity	Very Rapid and real‐time detection	Moderately rapid	Slow (dependent on the amount and antibody type and availability)	Rapid
Size	Smaller	–	Larger	Small
Reusability	Reversible	–	Gets denatured and Irreversible	Irreversible
Complexity	Ease to use	Labor intensive and technicians are required	Labor intensive	Ease to use
Turnaround time	Shortest	Longer	Short	Shorter
Stability	Highly stable but sensitive to nucleases in serum and real samples	Low stability	Low stability (sensitive to conditions)	Impaired sensitivity and stability at elevated temperature
Labeling and modification	Simple	Dependent on labeling	–	–
Point‐of‐care	Applicable	Applicable	Applicable	Applicable
Equipment requirement	No need for expensive equipment and reagent	Expensive equipment and reagent required	Expensive equipment and reagent required	No need for expensive equipment and can be done by non‐expert
Shelf‐life	Longer	–	Shorter	Longer
Commercialization	Problematic	Available in the market	Available in the market	Available in the market
Standardization	Standard protocol not available except *Pegaptanib* aptamer	Standard and universal protocol available	–	

The other significant problem for therapeutic aptamer is the delivery of nucleic acids into patients and the ability of nucleic acid aptamers to nucleases (Lakhin et al., 2013; Rozenblum et al., 2016). In addition, human blood nucleases have the potential to degrade unmodified DNA or RNA molecules. Despite these challenges, polyAAA tail modification and chemicals such as polyethylene glycol (PEG) and thymidine have been employed to improve their stability and longevity. However, these modifications may negatively affect aptamers' biosensing capability and increase the cost. Alternatively, these aptamers could be engineered into a circular form (Sun, Liu, Wei, et al., 2021) or delivered using nanoparticles (Figure 5b). Readers are referred to a recent review by Ni et al. (2020) for diverse chemical modification strategies for aptamers in relation to their stability enhancement and prolonged serum half‐life for intended therapeutic applications.

In addition, by conjugating these aptamers to biocompatible and/or medically relevant nanoparticles such as liposomes, quantum dots, fullerenes, viral‐like particles, among others, their stability, circulation features, and targeting specificity could be enhanced. Also, they can be delivered with lipid nanoparticles and structured RNA or DNA nanoparticles (Figure 5b). Although aptamers can be isolated or produced via chemical solid‐phase synthesis (in vitro SELEX) and machine learning techniques, specific in vivo conditions may be challenging to incorporate to enable their clinical translation. Due to this, oligonucleotide aptamer structure, affinity, and specificity may be impacted in actual samples. Recently, some novel high‐throughput methods for automatic SELEX approach and biosensor fabrication have been developed to resolve these challenges (Pan et al., 2018; Rozenblum et al., 2016).

Finally, cryo‐electron microscopy and crystallography studies on coronavirus aptamers are necessary to reveal their epitope landscape. This will enable the future rational combination of aptamers with non‐overlapping epitopes as potent anti‐SARS‐COV‐2 therapeutics. Unfortunately, only a few studies have used these tools to characterize the aptamer interaction with the spike glycoprotein (Kacherovsky et al., 2021).

## 8 CONCLUSION

In addition to public health protocols, diagnostics, therapeutics, and prophylactics form a relevant part of the toolkits in combating COVID‐19 outbreaks. These enable healthcare providers or settings to distribute resources and focus on patients with severe infections. Hence, preventing virus contagion and reducing the mortality rate. However, the recently available testing capacity and therapeutics cannot satisfy the extraordinary global demands for rapid, reliable, and extensively accessible molecular diagnostics and treatments. Hence, there is a need for further and/or alternative high‐throughput toolkits and effective treatments. Aptamers' unique characteristics and benefits, including easy synthesis, sensitivity, user‐friendly, higher stability, and modifications make them promising for developing novel therapeutics and diagnostics for viral infections with particular emphasis on coronaviruses. This will aid in the development of a reliable point‐of‐care detection and treatment technologies for SARS‐COV‐2 and other emerging coronaviruses. Research advances on aptameric sensors, technologies, and applications demonstrate that they can be employed to diagnose and screen current and future pandemics. The pandemic pathogens can be detected in a rapid, sensitive, and specific manner to curtail infection aggravation and transmission. Also, recent advances in neutralizing aptamers and related nanotechnologies provide a new direction for SARS‐COV‐2 treatment. Due to their abundance and relative conservation in coronaviruses, the nucleocapsid (N) protein, stem‐helix, and other nonstructural proteins could be possible targets for detecting, treating, and inhibiting the COVID‐19 and future coronavirus pandemic. These targets are less investigated but remain significant antiviral sites. Moreover, it is evident that integrated in silico methods and Artificial intelligence can be used to identify specific aptamer for improved aptasensing technologies and therapeutics against the SARS‐COV‐2, consequently speeding up the design and development of point‐of‐care testing and therapeutics to combat the pandemic.

## CONFLICT OF INTEREST

The authors have declared no conflicts of interest for this article.

## AUTHOR CONTRIBUTIONS

**Christian K. O. Dzuvor:** Conceptualization (lead); visualization (lead); writing ‐ Original draft (Lead); writing ‐ review and editing (equal). **Ebenezer Larteh Tettey:** writing – original draft – Supporting. **Michael K. Danquah:** Supervision (lead); visualization (equal); writing – review and editing (lead).

## RELATED WIREs ARTICLES

Application of nanotechnology in biosensors for enhancing pathogen detection

Advancements in protein nanoparticle vaccine platforms to combat infectious disease

Applications of nanotechnology in virus detection, tracking, and infection mechanisms

Aptamer‐functionalized hydrogels: An emerging class of biomaterials for protein delivery, cell capture, regenerative medicine, and molecular biosensing

Biomaterials and nanomaterials for sustained release vaccine delivery

## Data Availability

Data sharing is not applicable to this article as no new data were created or analyzed in this study.
